# Cytokines in CAR T Cell–Associated Neurotoxicity

**DOI:** 10.3389/fimmu.2020.577027

**Published:** 2020-12-16

**Authors:** Juliane Gust, Rafael Ponce, W. Conrad Liles, Gwenn A. Garden, Cameron J. Turtle

**Affiliations:** ^1^ Department of Neurology, University of Washington, Seattle, WA, United States; ^2^ Seattle Children’s Research Institute, Center for Integrative Brain Research, Seattle, WA, United States; ^3^ Shape Therapeutics, Seattle, WA, United States; ^4^ Department of Medicine, University of Washington, Seattle, WA, United States; ^5^ Department of Neurology, University of North Carolina, Chapel Hill, NC, United States; ^6^ Clinical Research Division, Fred Hutchinson Cancer Research Center, Seattle, WA, United States

**Keywords:** CAR T cell, ICANS, Cytokines, Neurotoxicity, Blood Brain Barrier (BBB)

## Abstract

Chimeric antigen receptor (CAR) T cells provide new therapeutic options for patients with relapsed/refractory hematologic malignancies. However, neurotoxicity is a frequent, and potentially fatal, complication. The spectrum of manifestations ranges from delirium and language dysfunction to seizures, coma, and fatal cerebral edema. This novel syndrome has been designated immune effector cell–associated neurotoxicity syndrome (ICANS). In this review, we draw an arc from our current understanding of how systemic and potentially local cytokine release act on the CNS, toward possible preventive and therapeutic approaches. We systematically review reported correlations of secreted inflammatory mediators in the serum/plasma and cerebrospinal fluid with the risk of ICANS in patients receiving CAR T cell therapy. Possible pathophysiologic impacts on the CNS are covered in detail for the most promising candidate cytokines, including IL-1, IL-6, IL-15, and GM-CSF. To provide insight into possible final common pathways of CNS inflammation, we place ICANS into the context of other systemic inflammatory conditions that are associated with neurologic dysfunction, including sepsis-associated encephalopathy, cerebral malaria, thrombotic microangiopathy, CNS infections, and hepatic encephalopathy. We then review in detail what is known about systemic cytokine interaction with components of the neurovascular unit, including endothelial cells, pericytes, and astrocytes, and how microglia and neurons respond to systemic inflammatory challenges. Current therapeutic approaches, including corticosteroids and blockade of IL-1 and IL-6 signaling, are reviewed in the context of what is known about the role of cytokines in ICANS. Throughout, we point out gaps in knowledge and possible new approaches for the investigation of the mechanism, prevention, and treatment of ICANS.

## Introduction

Neurotoxicity is one of the most common and dangerous complications of chimeric antigen receptor–modified T (CAR T) cell therapy (1). CAR T cell therapy utilizes patient T cells that are genetically modified to express a chimeric receptor consisting of an extracellular antibody fragment to bind the cancer target, and intracellular signal transduction domains. When the receptor binds the cancer target, it transduces a signal to the T cell to kill the target cell. Patients with refractory or relapsed hematologic malignancies have shown excellent responses to CAR T cell therapies (2), but toxicities such as cytokine release syndrome (CRS) and neurotoxicity continue to pose clinical challenges.

While neurotoxicity is apparently fully reversible in most cases, fatal cerebral edema and other life-threatening complications such as seizures and coma continue to occur both in clinical trials and with commercial CAR T cell products (3, 4). Because a variety of cell-based immunotherapies have been associated with neurologic adverse effects, this novel neurologic syndrome was designated “immune effector cell–associated neurotoxicity syndrome” (ICANS) by the American Society for Transplantation and Cellular Therapy (ASTCT) toxicity consensus group (5). For the purpose of this review, we will preferentially use “neurotoxicity” when discussing proposed disease mechanisms, and “ICANS” when referring to the clinical syndrome, with the understanding that this review focuses on CAR T cell mediated neurotoxicity and only makes brief mention of other immune effector therapies.

The role of secreted inflammatory mediators has been of great interest in efforts to understand the pathophysiology of ICANS. Systemic cytokine release and the severity of CRS are the most clearly defined risk factors for ICANS, as reported in multiple clinical trial cohorts in children and adults, in B cell leukemia and lymphoma, and with both CD28 or 4-1BB costimulated CAR T cell products (6–13). Therefore, a detailed examination of the role of the inflammatory secretome in the development of neurotoxicity may shed light on the pathophysiology of this still poorly understood complication (1).

In addition to direct effects of proinflammatory mediators on the CNS, there may be a contribution of cellular toxicity, such as from infiltrating T cells, macrophages, or other cell types. To date, there is no direct evidence that CD19, CD22, or BCMA-directed CAR T cells cause on-target, off-tumor toxicity in the CNS due to the CAR binding antigen on normal tissue.

In this review, we will examine the following key questions, as summarized in **Table 1**:

1. *Which secreted mediators are associated with neurotoxicity?* Consistent association between specific inflammatory mediators and neurotoxicity risk may suggest the highest-yield candidates for further investigation. Here, we will discuss the current evidence for involvement of both cytokines and other secreted factors such as angiopoietins in ICANS pathogenesis, and their contribution to other neuroinflammatory conditions that may have mechanistic pathways in common with neurotoxicity,
2. *Which cells secrete these inflammatory mediators?* It is clear that CAR T cells do not act alone, and activated endothelium and monocytes/macrophages have also been implicated in ICANS-related cytokine production. We will examine evidence for and against a direct role of cellular effectors during neurotoxicity,
3. *How do inflammatory mediators act on the CNS?* Cytokines have potential effects on many components of the CNS, including the neurovascular unit, which shields the brain from circulating effectors. Alterations in cytokine signaling during neurotoxicity may results in direct cytotoxic effects, or alterations in glial solute handling, neuronal excitability, neurotransmitter production, and cerebral perfusion. Understanding the response to systemic inflammation by individual cell types in the CNS will enable us to make sense of the complex and often contradictory data in the literature,
4. *How can this pathophysiologic process be modulated to improve clinical outcomes?* The complex web of possible neurotoxic interactions makes it challenging to predict which components could be modulated to alter the system in such a way to still allow full CAR T cell efficacy without causing neurologic dysfunction. The most informative approach right now is to critically examine clinical and nonclinical data for evidence of efficacy in modulating neurotoxicity, and design rigorous clinical interventional studies that can be integrated with both research and commercial CAR T treatment. In addition, we will discuss putative areas of intervention that have not yet been attempted in the clinic.

**Table 1:** Key points for cytokines and effector cell types.

Topic	Key Points
Mediators likely or possibly associated with ICANS	**IFN**γ, **IL-15**, **IL-6**, **IL-10**, **GM-CSF**, IL-2, IL-2Rα, **IL-1RA**, CXCL10, Granzyme b
Mediators likely NOT associated with ICANS	IL-4, IL-5, IL-7, IL-13, **Ang-1**, perforin
Mediators with indeterminate ICANS association	**Ang-2**, **Ang2/1 ratio**, CRP, EGF, eotaxin, FLT3L, Fractalkine, GCSF, GRO, Granzyme a, IFNα, IL-17, IL-18, IL-1α, **IL-1β**, IL-2, IL-22, IL-3, IL-8, MCP-1, MIG, MIP-1a, MIP-1b, PIGF, sIL6R, SVCAM1, TGFβ1, TNF, TNFRp55, TNFRp75, VEGF
CSF cytokines	Most commonly similar to serum levels
IL-15	Regulates T cell effector function Can exacerbate neuroinflammation and cerebral edema in animal models
IFNγ	Released by proliferating CAR T cells Complex regulator of CNS response in viral infection and autoimmunity
IL-1β	Produced by monocytes/macrophages, processed in inflammasome Key mediator of CNS response to injury, inflammation, and neurodegeneration
GM-CSF	Myeloid proliferation and activation, CD4^+^ T cell activator
IL-6	Key mediator of CRS Modulates CNS response to injury
Angiopoietin-Tie2 axis	Increased Ang-2 signaling activates endothelial cells May provide link between cytokine release and endothelial dysfunction
IL-10	Primary role as anti-inflammatory counter regulator, but may also have pro-inflammatory effects in the CNS.
CAR T cells	CAR T cells can produce most ICANS-associated cytokines
Monocytes/macrophages	CAR T cells can induce monocyte/macrophage inflammatory responses, which can be amplified by autocrine feedback loops
CNS resident cells (endothelium, pericytes, microglia, astrocytes)	Can respond to and produce many ICANS-associated cytokines

Bolded secreted mediators are discussed in detail in the text.

## Clinical Phenotype of Neurologic Dysfunction in CAR T Cell Therapy

To begin, we will review the typical clinical presentation and impact of ICANS to emphasize the features that may shed light on a pathophysiologic understanding from a cytokine perspective. Comprehensive reviews of clinical presentation, findings on clinical studies such as brain imaging, CSF examination, and electroencephalography (EEG), as well as toxicity grading and interventions, are available elsewhere (1, 14–18).

### Incidence of ICANS

**Figure 1:**
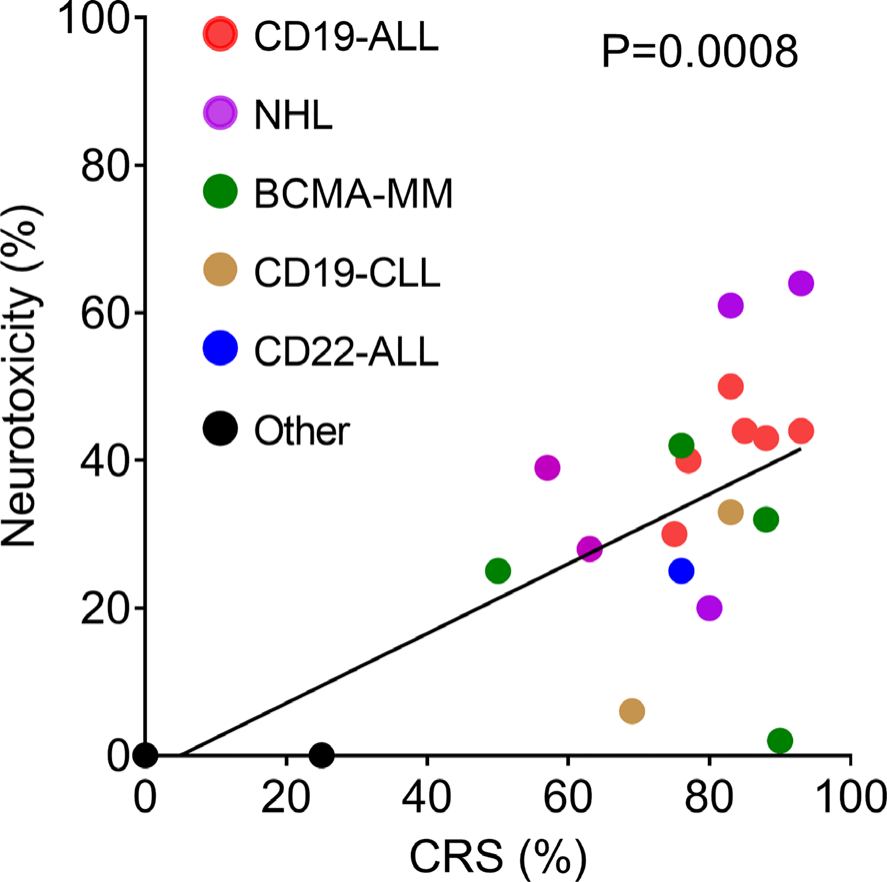
Correlation of incidence of neurotoxicity and CRS reported by clinical trials listed in **Table 2**. The solid line shows the linear regression, R^2^ = 0.4523.

Neurologic adverse events have been reported for all CAR T cell products with definitive clinical efficacy in hematologic malignancies (**Table 2**). This includes CAR T cells directed against CD19 [for acute lymphoblastic leukemia (ALL), non-Hodgkin lymphoma (NHL), and chronic lymphocytic leukemia (CLL)]; CD22 (to treat ALL); and B cell maturation antigen (BCMA) to treat multiple myeloma. Rates of ICANS vary from as low as 2% to as high as 60%–70% (10, 14, 37). For ease of comparison, we have plotted the reported rates of ICANS as a function of CRS for all published clinical studies that provided this information (**Figure 1**). Much of the variability in rates of ICANS can be explained by variability in CRS, where studies with lower rates of CRS also have less ICANS. However, some trials report much higher or lower rates of ICANS than can be explained by CRS alone. Some of this additional variability might be accounted for by differences in grading schemes. Most of the pivotal CAR T cell trials employed the Common Terminology Criteria for Adverse Events (CTCAE) toxicity criteria, whereas more recent investigations have utilized the ASTCT consensus ICANS grading scheme (5). Other causes for variability likely include patient population characteristics, properties of individual CAR constructs, and the type of malignancy treated.

**Table 2:** CAR T cell clinical trials with reported incidences of CRS and neurotoxicity.

	CAR construct	CRS (%)	sCRS (%)	ICANS (%)	sICANS (%)
**ALL**					
Grupp et al. (19)	CD19 4-1BB^t^	–	13^A^	–	9^A^
Park et al. (20)	CD19 28z	85^M^	26	44^C^	42
Maude et al. (9)	CD19 4-1BB^t^	77^P^	46	40^C^	13
Fry et al. (21)	CD22 4-1BB	76^C*^	0	25^C^	0
Gardner et al. (22)	CD19 4-1BB	93^C*^	23	44^C*^	21
Turtle et al. (23)	CD19 4-1BB	83^C^	23	50^C*^	50
Lee et al. (24)	CD19 28z	75^C*^	30	30^C^	5
Maude et al. (25)	CD19 4-1BB^t^	88^C*^	27	43^C^	–
**NHL**					
Brudno et al. (26)	hCD19 28z	80^A^	10	20^C^	5
Pasquini et al. (4)	CD19 28z^a^	83^A^	14	61^A^	–
Jaglowski et al. (3)	CD19 4-1BB^t^	–	4^A^	–	4^A^
Kochenderfer et al. (27)	CD19 28z	–	18^C^	–	55^C^
Neelapu et al. (10)	CD19 28z^a^	93^L^	13	64^C^	28
Schuster et al. (28)	CD19 4-1BB^t^	57^P^	18	39^C^	11
Turtle et al. (29)	CD19 4-1BB	63^C*^	13	28^C*^	28
**CLL**					
Fraietta et al. (30)^#^	CD19 4-1BB	69^C^	38	6^C^	0
Turtle et al. (31)	CD19 4-1BB	83^C*^	8	33^C*^	25
**Hodgkin Lymphoma**					
Ramos et al. (32)	CD30 28z	0^C^	0	0^C^	0
Wang et al. (33)	CD30 4-1BB	0^C^	0	0^C^	0
**Multiple Myeloma**					
Raje et al. (34)	BCMA 4-1BB	76^L^	6	42^C^	3
Cohen et al. (35)	BCMA 4-1BB	88^P^	32	32^C^	12
Brudno et al. (36)	BCMA 28z	93^C^	29	–	14^C^
Zhao et al. (37)	BCMA 4-1BB	90^L^	7	2^C^	0
Ali et al. (38)	BCMA 28z	50^C^	25	25^C^	8
**AML**					
Ritchie et al. (39)	LEY 28z	25^C^	0	0^C^	0

sCRS, severe CRS (grade ≥ 3). sICANS, severe ICANS (grade ≥ 3). CRS and ICANS grading systems used by each trial is denoted by superscripts as follows (15): A, ASTCT criteria; C, CTCAE criteria; L, Lee criteria; P, PENN/CHOP criteria; M, MSKCC criteria; *, modified, please refer to the individual publication for details. -, not reported. ^#^, toxicities only reported for responders.

For CAR T cells directed against solid tumors, the cytokine-related toxicity profiles are not as well defined. Brain tumor-directed CAR T cells have been associated with transient neurologic events such as seizures and focal weakness (40, 41), which may be more likely related to local brain inflammation and edema, rather than a systemic response. There are no published reports to date of neurologic adverse events during treatment with CAR T cells for non-CNS solid tumors. One possible explanation is the fact that CAR T cell proliferation and cytokine release require target recognition. Robust anti-tumor activity remains elusive in solid tumor CAR T cells, and consequently limited CAR T cell proliferation and cytokine release take place.

Immune effector cell engaging therapies can cause neurologic toxicity even in the absence of adoptive cell transfer. Blinatumomab, a bispecific CD3/CD19 T cell engager used in treatment of ALL, can cause severe CRS, and neurotoxicity occurs in 40%–50% of patients (42, 43). The most common manifestations are tremor, dizziness, paresthesia, and confusion (44). In contrast to CAR T cells, the presence of CRS does not appear to increase the risk of neurotoxicity. In a cohort of 225 patients treated with blinatumomab, the incidence of neurotoxicity was 58% in patients with CRS, and 51% in patients without CRS (44). Blinatumomab induces transient rises in cytokines, including IL-2, IL-6, IL-10, IFNγ, and TNF, which are most pronounced in the first cycle when neurologic symptoms are also most common (45, 46). Blinatumomab can induce endothelial activation as evidenced by increased serum angiopoietin-2 in treated patients, and *in vitro* can cause increased adhesion of T cells to endothelial cells (47).

### Time Course of ICANS

The typical ICANS time course is monophasic, with symptoms quickly ramping up to maximum and then improving over time, although waxing and waning can occur. Rapid development of ICANS symptoms is commonly observed in cases of fatal cerebral edema, where patients can go from being neurologically normal to dying from brain herniation within 24 h. Neurologic signs and symptoms typically begin 3–6 days after CAR T cell infusion, with the peak of symptoms around day 7 or 8, and resolution by days 14–21 (7, 8, 11, 12). Severe ICANS symptoms are more frequently observed in cases when CRS develops early (7), which may be due to a high dose of CAR T cells, or unusually robust and rapid CAR T cell proliferation. Neurologic adverse events typically occur after the onset of CRS, and it is not unusual for ICANS to develop in the setting of improving or resolved CRS. This observation supports the hypothesis that cytokine release contributes to the development of neurotoxicity, with a lag of several days between the peak of cytokine levels and the peak of neurologic symptoms. ICANS can also develop in the absence of CRS, although this is less common (1, 12).

### Signs and Symptoms

To understand the pathophysiology of ICANS, it is important to consider whether there are parallels between CAR-T cell related neurotoxicity and other neurologic sequelae that occur with systemic immune activation. Overall, the constellation of typical signs and symptoms appears to be relatively specific to ICANS, and especially neurotoxicity after CAR T cells for hematologic malignancies. The majority of experience has been in patients treated with CD19-directed CAR T cells, but the syndrome appears similar in BCMA- and CD22-targeted CAR T therapies.

The most prevalent ICANS symptom by far is transient cognitive impairment. This is variably described by authors as confusion, delirium, or encephalopathy, and predominates in both children and adults, as well as in patients with different underlying hematologic malignancies (6–8, 12, 48). Cognitive dysfunction is often associated with a striking phenotype of language dysfunction, ranging from word finding difficulty to mutism. Even though language and/or handwriting disturbance affects the majority of patients who develop ICANS, it is almost never associated with focal abnormalities on MRI that would implicate injury localized to recognized language areas of the brain.

Although tremor and headache are relatively nonspecific and are thus not considered core symptoms of ICANS for diagnostic purposes, they commonly coexist with or precede more definitive neurologic impairment. Altered level of consciousness occurs in more severe cases, and can lead to coma and requirement for mechanical ventilation. Seizures, both clinical convulsions and electrographic seizures without a motor correlate, occur in upward of 5%–10% of patients, with incidence varying from 0% to 30% after CD28-costimulated CD19-CAR T cells, and 3%–14% with 4-1BB CD19-CARs (14). Focal neurologic symptoms are less common, although EEG commonly shows focal abnormalities (11, 49, 50). Rarely, cortical cytotoxic edema seen during acute ICANS can evolve into chronic injury with persistent focal dysfunction, and evidence of gliosis on histopathology (7, 8).

The most feared neurologic complication is cerebral edema, which is fatal in most cases. Although comprehensive data is currently not available, we estimate that 1%–2% of CD19-CAR T–treated patients develop cerebral edema based on the reported incidence across clinical trials. Cerebral edema has not been described with other CAR T cell products or other cancer immunotherapies. Cerebral edema has occurred in children and adults, in patients with ALL, NHL, and CLL, and in CD28 and 4-1BB costimulated CAR T cell products (7, 28, 51–54). Many of the hypotheses on ICANS pathophysiology are based on findings in patients with severe or fatal neurotoxicity, such as microvascular disruption, endothelial activation, and brain edema. However, more research is needed to determine whether these are present to a milder degree in reversible ICANS, or whether the pathophysiologic mechanisms are distinct.

### Imaging

Imaging findings in ICANS have some striking similarities with MRI patterns seen during CNS inflammatory conditions, and some types of CNS infections.

The first pattern that can be seen in all three is leptomeningeal enhancement and T2 hyperintensity in the cerebral sulci (7, 11), an imaging appearance which is suggestive of infectious or inflammatory meningitis. However, CSF cell counts are typically only mildly elevated during ICANS (1). An explanation of this finding may be an opening of the meningeal blood-CSF barrier, with meningeal vessels becoming more permeable (55).

The second finding that can be seen in ICANS, CNS inflammation, or CNS infection is T2 hyperintensity and swelling of the bilateral thalami, indicative of interstitial or vasogenic edema (8, 12). This can be associated with diffusion restriction in the same area, which suggests additional cytotoxic edema. This pattern of symmetric thalamic change is quite common in multiple neuroinflammatory conditions, including acute demyelinating encephalomyelitis (ADEM) (56), acute necrotizing encephalopathy of childhood (ANEC) (57), and arboviral encephalitides (58). In addition, the thalami are commonly affected in a number of neurometabolic derangements, including hypoxia and mitochondrial dysfunction, and in thrombotic microangiopathies such as hemolytic-uremic syndrome (59). It is unknown what causes the specific susceptibility of this brain region, but it may be due to differences in energy demand or microvascular blood supply. The brainstem, especially the medulla, can also demonstrate interstitial or cytotoxic edema in severe cases of ICANS, typically associated with concomitant changes in the thalami and other deep gray matter structures.

Other common patterns of MRI abnormalities during ICANS include symmetric T2 hyperintensities in the supratentorial white matter, diffusion restriction in patchy areas or cortex and/or white matter, and reversible interstitial edema in areas of prior CNS injury such as from radiation or medication toxicity (7, 8).

In conclusion, CNS imaging changes are typically symmetric, and have a predilection for uniquely susceptible brain regions, such as the thalami and deep gray matter. This suggests that ICANS may be triggered by a systemic process, such as systemic cytokine elevation, and that it may engage final common pathways that are active during other types of CNS infection or inflammation.

### CSF Analysis

The interpretation of CSF measurements in ICANS has been hampered by the fact that CSF is rarely obtained from CAR T recipients without ICANS during the 10–14 days following CAR T cell treatment (the typical time frame for ICANS development). Therefore, it is not known whether the findings during acute ICANS would also occur in patients who have CAR T cell expansion but no ICANS.

CAR T cells are found in the CSF of most patients who have had successful CAR T cell expansion. In a pediatric study of CD19-CAR T cell therapy for ALL, all subjects underwent lumbar puncture on day 21 after CAR T cell infusion (8). CAR T cells were found in the CSF of all patients with ICANS, but also in 90% of patients without neurological symptoms. These findings suggest that trafficking of CAR T cells into the CSF space is not a primary mechanism of neurotoxicity.

CSF protein is frequently very abnormal during ICANS, with median levels of 80–110 mg/dl, and occasional values over 1,000 mg/dl (7, 8, 11, 12). The CSF/serum albumin quotient is increased, and higher protein levels were associated with more severe neurotoxicity (12). The composition of the elevated CSF protein has not been reported in the literature, and it is therefore unknown whether it represents mainly leakage of serum proteins through a permeable blood-CSF barrier, or additional local synthesis in the CNS. Levels of cytokines in the CSF are frequently quite elevated (see below), but usually mirror the levels found in the serum, with only some studies reporting elevation above serum levels for some analytes. These data suggest that there is increased access of the systemic secretome to the CNS *via* the blood-CSF barrier.

## Secreted Mediators Associated With Neurotoxicity in Immunotherapy

### Patient Cytokine Profiles

**Figure 2:**
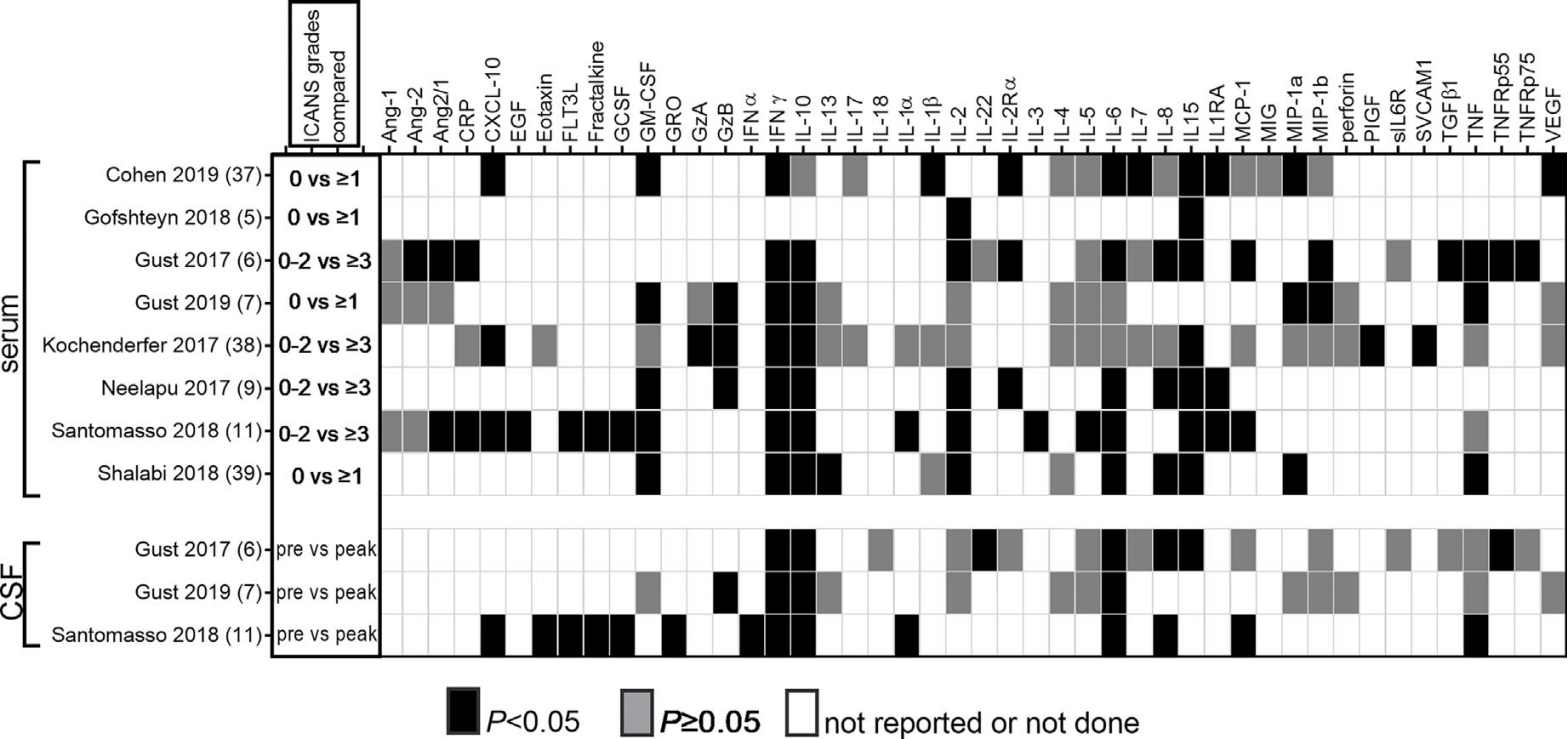
Reported associations of serum and CSF cytokine levels with ICANS. All published studies that reported association of cytokine measurements with ICANS are summarized. The first column shows the ICANS grades that were compared to determine whether there was an association of cytokine levels with either presence or absence of ICANS in half of the studies, or between none/mild and severe ICANS in the other half. In studies that reported CSF cytokine measurements, baseline samples were compared to those obtained during acute ICANS.

Cytokine measurements in the serum/plasma and CSF were performed in many of the pivotal clinical trials that established the efficacy of CAR T cell treatment. We conducted a comprehensive literature search to identify all published clinical trials of CAR T cells that measured levels of secreted mediators in serum and/or CSF during the 21-day period following CAR T cell infusion, and who reported whether or not these were associated with risk of ICANS. We identified 8 studies that reported cytokine associations with ICANS (6–8, 10, 12, 27, 35, 60). These studies employed a variety of cytokine panels that were evaluated using either bead-based or electrochemoluminescence assays. The findings from these studies are summarized in **Table 1** and **Figure 2**.

### Important Caveats for Interpreting Clinical Cytokine Profiles in ICANS

When interpreting clinical cytokine measurements, several limitations to the data must be considered: the confounding effect of concurrent CRS, and variability in ICANS grading, comparison groups, cytokine panel selection, timing of samples, and cytokine-directed interventions.

The most important obstacle to identifying neurotoxicity-specific cytokines and other signaling molecules is the fact that CRS is often present simultaneously. This may obscure the importance of some markers, while others may appear strongly associated with ICANS when they are actually more reflective of the higher rates of concurrent CRS in patients with ICANS. Some investigators have attempted to control for the presence of concurrent CRS by reporting cytokine elevations that were exclusively associated with ICANS but not CRS, although methodologies for comparison and results have been inconsistent. In a pediatric study of CD19-CAR T cells, 28 of 43 cytokines on a panel were associated with ICANS. However, only IL-2, soluble IL-4 receptor, hepatocyte growth factor, and IL-15 were uniquely elevated in patients with neurotoxicity, but not in patients with CRS alone (6). In a study of BCMA-CAR T cells for multiple myeloma, CXCL10 and IL-7 were the only cytokines that were elevated in ICANS but not CRS (35). In another pediatric CD19-CAR study, patients were stratified by CRS grade to account for the confounding effect of CRS. IL-10 and granzyme B, but not IL-6 and IFNγ, were significantly higher in patients with ICANS compared to those who had the same CRS grade but no ICANS (8). However, most investigators have not attempted to control for the presence of CRS, and therefore, we are summarizing reported associations of secreted markers with neurotoxicity irrespective of whether the patients may have had concurrent CRS.

Variability of ICANS grading and assignment to comparison groups may further complicate the interpretation of cytokine measurements, rendering it impossible to directly compare the absolute levels of cytokines between studies. For example, some investigators compared patients with no ICANS against those with any grade of neurological symptoms, while others compared mild versus severe ICANS, or association of specific cytokines with specific neurologic signs and symptoms. Therefore, for the remainder of the review, we have adopted a simple categorization of “associated with ICANS” versus “not associated with ICANS”.

An additional concern is that potentially important inflammatory mediators were not measured by the majority of studies. The reported cytokine panels were typically designed to better understand the signaling environment that supports CAR T cell proliferation and anti-tumor activity, rather than being neuroinflammation-specific. Further, some authors report their findings for all tested cytokines, whether they were significantly associated with ICANS or not, while others report only the statistically significant associations. This may lead to an under-reporting of markers that are consistently found to NOT be associated with ICANS, hampering our ability to identify pathways that are unlikely to contribute to ICANS. Despite these caveats, consistent patterns emerge from the literature.

### Serum Cytokines

Increased serum levels of IFNγ and IL-15 were associated with ICANS in every study that investigated these cytokines. Other markers with significant association in a majority of studies include IL-6, IL-10, GM-CSF, IL-2, IL-1RA, and CXCL10. A mixed pattern emerges for IL-1β, IL-8, and TNF. Consistent lack of association was found for a few cytokines, for example, IL-4 levels were not associated with ICANS in any of the 4 studies that reported them. For the majority of other markers, it is difficult to draw conclusions because they have been investigated by few studies.

### CSF Cytokines

Since levels of inflammatory mediators in the CNS parenchyma cannot be directly measured in patients, CSF levels are frequently used as a proxy. Molecules are trafficked across the epithelial barriers of the choroid plexus into the CSF (61), which is distinct from the active transport of cytokines from the blood into the brain parenchyma *via* the neurovascular unit (62). Systemic inflammation may alter the permeability of these barriers to cytokines (63), and induce additional local cytokine production in the CNS by infiltrating immune cells and CNS resident cell types (discussed below).

Three studies reported cytokine analyses in the CSF (7, 8, 12). All reported comparisons between CSF cytokine levels before and after CAR T cell infusion, and between patients with different degrees of ICANS, but not between patients with and without ICANS. This is due to the unavailability of control CSF during the acute post-infusion phase from patients who did NOT develop ICANS. Therefore, we do not know whether elevation of CSF inflammatory markers above baseline is related to the pathophysiology of ICANS, or whether it may simply represent spillover of serum cytokine levels. To address this question, all three studies compared acute CSF and serum cytokine levels to determine whether there might be local cytokine synthesis in the CNS. In most cases, CSF cytokine elevations during acute neurotoxicity mirrored those in the serum (7, 8, 12). Enrichment of IL-8, CXCL10 and MCP-1 was seen in the CSF compared to the blood in some patients with severe neurotoxicity (12), but others found no cytokines that were higher in the CSF than in the serum (7, 8).

### Cytokine Kinetics

The timing of cytokine production is tightly linked to the kinetics of CAR T cell expansion. Importantly, the peak of CRS is approximately 1 day before the peak of ICANS, and both occur prior to the peak of peripheral blood CAR T cell counts (7, 64). In the blood, CAR T cell counts typically rise from less than 1 cell/μl on day 3 to 100–1,000 s of cells/μl blood on day 8–14 after infusion, with the steepest slope of increase occurring around day 5 (65). After the peak, the CAR T cell numbers contract again, although they may remain detectable in the peripheral blood for months and years (20).

Only two studies have reported detailed data on the association of cytokine kinetics over time with neurotoxicity (7, 12). The peak levels of serum cytokines that are associated with increased neurotoxicity risk typically occur around days 5–7 after CAR T cell infusion, when CAR T cell doubling rate is the fastest. Exceptions include IL-2 and MCP-1, which peaked earlier, between days 1 and 3 (7, 12).

Several investigators have attempted to identify early markers of toxicity risk, which would allow risk stratification of patients and preventive treatment with cytokine blockade. In patients with severe neurotoxicity, IL-6, IFNγ, IL-2, IL-10, IL-15, and MCP-1 were already significantly higher within the first 36 h after CAR T cell infusion in both studies reporting these time points (7, 66). Results have been less consistent when trying to identify cytokines that predict neurotoxicity risk prior to lymphodepleting chemotherapy and CAR T cell infusion. One study reported elevations of several cytokines, including IL-6 and GM-CSF, pre-therapy in patients who would go on to develop severe neurotoxicity (66). Other studies have not found the same association (7, 8).

### IL-15

IL-15 is one of the most consistently identified systemic and CSF markers of ICANS. All 6 studies that measured serum concentrations of IL-15 showed a correlation with ICANS (**Figure 2**). IL-15 concentration in the CSF was higher during ICANS than at baseline in the one study in which it was reported.

IL-15 plays a key role in T cell effector functions, and is used during generation of CAR T cells to induce proliferation and activation (67). Higher baseline or peak serum IL-15 levels have been associated with better anti-tumor responses after CD19-CAR T therapy, but also higher rates of severe ICANS and CRS (27, 68). It is possible that the association of IL-15 with ICANS is a bystander effect, where higher IL-15 levels are key for robust CAR T cell proliferation, thereby increasing the risk of CRS and ICANS without a direct role of IL-15 on the CNS. However, there is some evidence that IL-15 can cause neurotoxicity. Treatment of non-Hodgkin lymphoma with lymphodepletion followed by haploidentical natural killer cell-enriched donor cells and recombinant IL-15 was associated with neurotoxicity in 38% of patients. Interestingly, neurotoxicity and CRS only occurred if the IL-15 was given subcutaneously, but not if administered intravenously (69, 70). IL-15 is expressed by tissues as a danger signal that communicates to the immune system that the tissue is under attack. It provides co-stimulation to cytotoxic T cells to instruct them to eliminate infected cells (71). In mice, IL-15 induces cytokine production in microglia, which could amplify the local inflammatory response in the CNS (72). Increased IL-15 expression in astrocytes led to worsening of cerebral edema in mice subjected to experimental intracranial hemorrhage (73). Thus, overactive IL-15 signaling is a plausible contributor to ICANS. Unfortunately, blockade of IL-15 signaling might increase the risk of an unsatisfactory antitumor response.

### IFNγ

IFNγ is a key cytokine that participates in the regulation of complex networks of soluble mediators in viral pathogen response and autoimmunity (74–76). Increased serum IFNγ levels during the acute post-CAR T phase were reported as associated with ICANS by every study we evaluated (**Figure 2**). In addition, all 3 studies that measured CSF levels found IFNγ increases above baseline in patients with ICANS.

IFNγ is released by proliferating CAR T cells, and production of IFNγ and other pro-inflammatory cytokines differs between types of CAR T cell therapies. In patients treated with a CD19-CAR T cells incorporating a fully human CAR, lower IFNγ production by the CAR T cells *in vitro* was associated with lower serum levels of IFNγ and decreased rates of ICANS, compared to T cells modified with a non-humanized CD19-CAR (26).

Preclinical studies paint a complex picture of the regulatory role of IFNγ in CNS inflammation. IFNγ mediates activation of infiltrating immune cells such as T cells, monocytes/macrophages, and natural killer cells (75), and is also responsible for microglial activation and antigen-presenting function (77). In endothelial cells, IFNγ modulates gene expression (78) This can, for example, alter leukocyte adhesion to the endothelium by decreasing expression of E-selectin, while increasing ICAM-1 and VCAM-1 (79).

The *in vivo* effects on CNS inflammation are likely regulated by an interplay of multiple cell types at the neurovascular unit (80). In mice, IFNγ is required for trafficking of T cells and monocytes into the CSF *via* the choroid plexus (81). In rats, direct injection of IFNγγinto the brain caused demyelination, whereas systemic administration of IFNγ protected against demyelination in animals with experimental autoimmune encephalomyelitis (82). IFNγ can stabilize brain endothelial tight junctions and prevent infiltration of leukocytes into the brain in experimental autoimmune encephalomyelitis (83), but conversely causes BBB leakage during reovirus infection (84). In addition, the effects of IFNγ differ by brain region, likely due to differential effects on infiltration of encephalitogenic T cells (82).

### IL-1β

IL-1β signaling is a key mediator of CNS response to injury, inflammation, or neurodegeneration (85). It is primarily produced by monocytes and macrophages and processed in the inflammasome. In human disease it is considered a mediator of autoinflammation that does not involve T-lymphocyte mediated autoimmunity (86). Unfortunately, only three clinical CAR T studies included IL-1β or the IL-1 receptor antagonist (IL-1RA) on their reported analyte panels. While IL-1RA was associated with ICANS in all three studies, only one of three studies noted an increase in IL-1β (**Figure 2**). No CSF data are available in CAR T cell patients. Despite these equivocal associations, there has been strong interest in exploring IL-1 blockade as a strategy for preventing ICANS. One rationale is the fact that IL-1 blockade is helpful in macrophage activation syndrome and hemophagocytic lymphohistiocytosis, which share many clinical and laboratory features with CRS (87, 88). The IL-1 receptor antagonist anakinra has excellent CSF penetration, and was able to reduce CSF IL-6 and TNF levels in patients with neonatal-onset multisystem inflammatory disease (89). The second rationale comes from a key preclinical study (90), which showed a direct role for IL-1 in CAR T cell related neurotoxicity in a humanized mouse model. After treatment with human CD19 CAR T cells, SGM3 mice developed generalized paralysis and seizures, which could be prevented by anti-IL-1 pretreatment. In this mouse model, neurologic changes did not develop until 28–30 days after CAR T treatment when CRS had long resolved, and was characterized by meningeal infiltration of human monocytes but no CNS parenchymal changes. IL-1β production by CNS-infiltrating neutrophils and monocytes is required for the development of experimental autoimmune encephalomyelitis, which can be partially replicated by injection of IL-1β (91). Brain endothelial cells express the IL-1 receptor 1, and IL-1β activity is amplified by a paracrine loop between infiltrating immune cells and the brain endothelium (92). Results of clinical trials of IL-1R antagonism (discussed further below) will be highly informative in guiding clinical practice.

### GM-CSF

GM-CSF was associated with ICANS in five of six studies in serum, but did not increase during ICANS in the CSF in the single study that reported CSF measurements (**Figure 2**). A putative role for GM-CSF in the development of neurotoxicity is supported by animal studies. Although GM-CSF is largely responsible for myeloid cell proliferation and activation, it is also a critical mediator of autoimmune or inflammatory activity of CD4^+^ T cells. CNS-infiltrating helper T cells secrete GM-CSF, which then activates microglia, leading to further secretion of pro-inflammatory mediators by the microglia (93). In a xenograft study, mice implanted with primary ALL blasts developed hunched posture, motor weakness and CRS after treatment with human CD19-directed CAR T cells (94). The mice also had increased contrast enhancement on brain MRI, which was ameliorated by concurrent treatment with the GM-CSF neutralizing antibody lenzilumab.

### IL-6

IL-6 was associated with ICANS in five of seven studies in serum, and increased in CSF from baseline in all 3 studies that measure it (**Figure 2**). A causative role for IL-6 in ICANS is not established, although its importance in CRS certainly makes it deserving of further study. Current data support a key role of IL-6 release in the pathogenesis of CRS, based on correlation of markedly elevated IL-6 levels with signs and symptoms of CRS (13, 64), as well as observational evidence of rapid resolution of CRS after administration of the IL-6 receptor blocker tocilizumab (95). However, the role of IL-6 in mediating neurotoxicity remains uncertain, based on clinical evidence that IL-6 blockade may not prevent the development of ICANS. Observational studies (further discussed below) reported equal or higher rates of ICANS when IL-6 blockade was used preemptively (8, 53, 96). In addition, clinical experience shows that tocilizumab typically fails to induce rapid improvement of established ICANS (7, 8).

IL-6 plays an important role in the response to CNS insults such as stroke and neurodegenerative disorders (97). It induces proliferation of microglia (98), and IL-6 deletion in mice leads to impaired neuroglial response to injury (99). The widespread use of tocilizumab for management of CRS provides an excellent opportunity for correlative clinical studies of its effect on ICANS.

### Angiopoietin-Tie2 Axis

Endothelial activation may provide a link between systemic cytokine release and CNS microvascular dysfunction. Indeed, the angiopoietin (Ang)-Tie 2 system has been implicated in ICANS (7). The system maintains vascular quiescence when Ang-1 binds the Tie-2 receptor, and induces a proinflammatory endothelial activation phenotype when Ang-2 is released from endothelial cells and displaces Ang-1 from the receptor (100, 101). The ratio of serum Ang-2 to Ang-1 was increased in two of three CAR T studies, while none reported a significant association of Ang-1 levels, and only one noted a difference in Ang-2 levels in patients with and without ICANS (**Figure 2**). Although the role of the Ang-Tie2 axis in the pathogenesis of sepsis is well established (102), there is limited direct evidence for a role of the Ang-Tie2 axis in CNS inflammation or BBB dysfunction. In cerebral malaria, which is discussed in more detail below, the evidence for involvement of the Ang-Tie2 axis points toward a clear role in severe malaria, while a specific pathogenic role in cerebral malaria is not supported by clinical evidence to date. For example, an association of decreased Ang1, and/or increased Ang2, Ang2/1 ratio and Tie2, has been repeatedly demonstrated in clinical studies of severe malaria, but the subset of patients with severe malaria who have cerebral malaria is indistinguishable from those with severe malaria but no cerebral involvement (103, 104). In animal studies, however, manipulation of Ang-Tie2 signaling toward the quiescent Ang1-dominant state does appear to protect the BBB, both in models of cerebral malaria (103) and traumatic brain injury (105).

### IL-10

IL-10 was elevated in serum during ICANS in six of seven studies, and increased in the CSF during ICANS in all three studies that measured it (**Figure 2**). Nonetheless, the consistent activation of the IL-10 response in ICANS has not received much attention. This is likely due to the well-established role of IL-10 as an anti-inflammatory counter-regulator that suppresses cytokine production and terminates inflammatory responses (106). However, IL-10 can also lead to a paradoxical increase in IFNγ production (107), and stimulates the cytotoxic response of CD8^+^ T cells (108). The role of IL-10 in CNS inflammation is only beginning to emerge (109). It is produced by microglia and astrocytes, but supplemental administration or overexpression of IL-10 did not ameliorate neuroinflammation in animal studies (109). Suppression of IL-10 secretion may be beneficial in inducing a more robust CAR T response (110), but pegylated IL-10 has also been noted to improve CAR T expansion (111). In summary, although the activity of IL-10 may be largely as a counter-regulator that is important for maintaining a monophasic inflammatory response after CAR T cell infusion, it could also lead to induction of additional toxic responses, which deserve further study.

### Cellular Sources of ICANS-Associated Secreted Mediators

The source of cytokines in blood and CSF is incompletely characterized in human CAR T cell patients. Many of the cytokines associated with ICANS can be produced by a variety of immune cells, both endogenous and adoptively transferred, as well as non-immune cell types including endothelium and stromal cells. CAR T cells alone are capable of producing high concentrations of ICANS-associated cytokines upon antigen stimulation *in vitro*, including IFNγ, TNF, GM-CSF, IL-6, IL-2, IL-10, IL-13, IL12p70, and IL-8 (112). If CAR T cells are the primary source of toxic inflammatory mediators, then toxicity risk might be improved by genetically manipulating the CAR T cells’ ability to express these mediators, a technique that has been shown as proof of principle using CRISPR/Cas9 technology (113). *In vivo*, more complex interactions between CAR T cells and other components of the immune system are likely responsible for the spike in cytokine production during CAR T cell proliferation. Even though IL-6 is a key cytokine implicated in the pathogenesis of CRS, IL-6 transcripts were not detected in peripheral blood T cells collected from patients who had developed CRS after CD19-CAR T cell therapy. This was despite the fact that the cells had microscopic appearance consistent with CAR T cell activation (114). Monocytes/macrophages likely play a key role in the production of IL-6 and IL-1 during CRS and/or ICANS, as shown both in co-culture experiments (114) and *in vivo* with a humanized mouse model (90). The production of IL-6 and MCP-1 by monocytes was shown to be dependent on GM-CSF signaling by CAR T cells *in vitro* (115). Knockdown of GM-CSF in CAR T cells *via* TALEN repressed secretion of IL-6 and MCP-1 by monocytes, while IFNγ, TNF, IL-8, and IL-10 were unaffected. Once initiated, macrophage inflammatory responses can then be amplified by autocrine positive feedback loops *via* catecholamine signaling (116). This was shown to be relevant for the development of CRS in mouse models (117): CRS was ameliorated by inhibition of catecholamine signaling by atrial natriuretic peptide or methyltyrosine in mice transgenic for human myeloid supporting cytokines (SMG3 mice) treated with human CD19-CAR T cells, and in wild-type mice treated with murine CD19-CAR T cells.

Cytokine production by endothelial cells may also be relevant in ICANS, as indicated in an autopsy study of a patient with fatal CRS that was accompanied by mental status changes. Here, IL-6 was expressed by brain endothelial cells but not infiltrating T cells (118).

## Known Roles of ICANS-Associated Cytokines in Neuroinflammatory Disorders

To provide some insight into possible final common pathways of how systemic cytokine elevation is related to CNS inflammation and brain edema, we will examine several systemic inflammatory conditions that are associated with neurologic dysfunction. Apart from CNS infection, these are all systemic conditions that lead to diffuse brain dysfunction, with clinical and imaging characteristics, as well as systemic and CSF cytokine profiles, that are similar to patients with ICANS. Cerebral edema is associated with a variety of neuropathological conditions, including trauma, ischemic or hypoxic injury, infection, liver failure, and inflammatory disorders such as multiple sclerosis. Breakdown of the blood-brain-barrier is the suspected pathogenic mechanism, although much remains to be learned (119, 120). Where appropriate, we point out key differences between ICANS and other disorders, which may indicate a divergence in pathophysiologic mechanisms.

### Encephalopathy and Cerebral Edema During Systemic Infectious and Inflammatory Disorders

Sepsis-associated encephalopathy denotes the neurologic dysfunction that can be present during the severe systemic inflammatory state of sepsis, in the absence of direct CNS infection (121, 122). The proposed pathophysiology includes systemic cytokine release, endothelial activation, and BBB leakage (122). The disorder most often manifests as reversible delirium, but can also lead to seizures, coma, and associated long term neurocognitive impairment (123). In a study of ICU patients, higher levels of IL-8, IL-1RA, MCP-1, and IL-10 were associated with delirium (124). Animal studies suggest a role for impaired perfusion of cerebral microvessels during sepsis (125).

Systemic infections such as influenza can be associated with rapidly developing diffuse cerebral edema, an infrequent but devastating complication especially in younger patients (126). Elevations of IL-6, IL-10, and sTNFR1, but not IL-2, IL-4, and IFNγ,γwere associated with poor outcome in influenza-associated encephalopathy, with serum levels of cytokines usually higher than in the CSF (127, 128). Children with HHV-6 related encephalopathy had increased levels of serum IL-6, IL-10, sTNFR1, and CSF IL-6 and TNFR1 (129). The histopathologic appearance of influenza-related cerebral edema (Reye-like syndrome) is strikingly similar to cases of cerebral edema in ICANS, with generalized brain edema, plasma extravasation around cerebral vessels, and increased macrophages along the vessels. Similar fulminant cerebral edema can occur with macrophage activation syndrome, a disorder of systemic immune dysregulation that can lead to uncontrolled cytokine storm (130).

In summary, there is marked overlap between signs and symptoms, laboratory markers and imaging findings in sepsis-associated encephalopathy and ICANS. The population of patients with sepsis is likely more heterogenous than patients undergoing CAR T therapy with less opportunity for baseline assessments. Therefore, it will be highly informative to exchange ideas and findings between the two fields of investigation.

### Inflammatory Mediators in Cerebral Malaria

Cerebral malaria is another disorder that may provide clues to the pathophysiology of CAR T neurotoxicity. In a subset of patients, infection with *Plasmodium falciparum* causes diffuse encephalopathy and cerebral edema, with resultant high mortality (131, 132). One possible mechanism is sequestration of infected red blood cells in the cerebral microvasculature, where they adhere to the endothelium *via* the *P. falciparum* erythrocyte membrane protein (Pfemp) and impair brain perfusion (133). Retinopathy is a common feature of cerebral malaria that is likely due to microvascular dysfunction, a finding which has not been seen in patients with severe ICANS who had ophthalmologic exams (11). Endothelial activation has also been implicated in the pathogenesis of cerebral malaria. Systemic Ang-1 levels increase during recovery in patients with severe malaria, and systemically delivered Ang-1 improved survival in a mouse model of cerebral malaria (103).

The second proposed mechanism is cytokine-related CNS dysfunction and brain swelling, which is plausible because microvascular sequestration is not always observed in cases of fatal cerebral edema, and because pro-inflammatory cytokine elevations are common in severe malaria. Multiple clinical studies have examined the association of systemic cytokine levels with risk of cerebral malaria, although results have been inconsistent. Some studies reported that IFNγ, TNF, IL-1β, IL-4, IL-6, IL-8, IL-10, IL12p70, and/or CXCL10 were elevated in children with cerebral malaria compared to those with uncomplicated malaria (134–136), while others reported no difference in cytokine levels between the groups (137). The degree of brain swelling was independent of plasma levels of IL-1β, IL-6, IL-8, and IL-10 in children with cerebral malaria, while IL-12 and TNF were elevated in patients with more severe edema (138).

### Thrombotic Microangiopathy

Disturbances of brain microvascular perfusion can lead to neurologic dysfunction, cerebral edema, and death. Thrombotic microangiopathies, such as hemolytic uremic syndrome or thrombotic thrombocytopenic purpura, entail a cascade from endothelial activation, formation of microthrombi, and obstruction of capillaries with resulting dysfunction of organs including the kidneys, lungs, gut, and brain (139). This pathologic sequence can be affected by systemic cytokine release and therefore is an important consideration in ICANS (7).

Disordered regulation of von Willebrand factor (VWF) is a key feature in the pathogenesis of thrombotic thrombocytopenic purpura, and may be involved in other types of thrombotic microangiopathies (140). When endothelial cells are injured, ultralarge VWF multimers are released from endothelial Weibel-Palade bodies. The VWF multimers can remain adherent to the endothelium, where they serve as binding sites for platelets and leukocytes and induce microvascular thrombosis (141, 142). *In vitro*, IL-8 and TNF cause increased release of VWF strings, and IL-6 impairs their cleavage (143). In addition, TNF, IFNγ, and IL-4 impair the production of the protease ADAMTS13, which is key for cleaving VWF strings (144).

The presence of thrombocytopenia and increased Ang2/1 ratio in patients with severe CAR T neurotoxicity (7, 12) raises the question whether endothelial activation and resultant thrombotic microangiopathy may contribute to neurologic dysfunction in CAR T cell patients. However, associated organ dysfunction such as kidney failure typically does not occur concurrently with ICANS, and histopathologic findings and serum marker signatures consistent with endothelial dysfunction are not seen in all CAR T studies. This indicates either a microvascular pathophysiology that is distinct from other thrombotic microangiopathies, differences across patient populations, or the fact that CRS may be a driver of these findings (64). Brain microvascular thrombi rich in platelets and van Willebrand factor were observed in one patient with fatal cerebral edema after CAR T cell treatment (7), but were absent in another case with similar clinical presentation (54). In adult patients with ALL, NHL or CLL, ICANS was associated with elevations in serum VWF and IL-8, as well as thrombocytopenia, elevated D-dimer, and decreased fibrinogen levels, all indicative of coagulopathy (7, 12, 145). Interestingly, serum of patients with severe ICANS induced decreased formation of VWF strings on human umbilical vein endothelial cells, possibly due to depletion of available high molecular weight VWF (7). In contrast, in pediatric patients with ALL who received CD19-directed CAR T cells, there was no association of ICANS with serum markers of coagulopathy and/or endothelial activation, including VEGF-A, VWF, Ang-1, Ang-2, Ang-2/1 ratio, peak INR and D-Dimer, or nadir fibrinogen levels and platelet counts (8). In summary, a possible causative role of endothelial dysfunction and microangiopathy in ICANS remains to be clarified in further studies.

### CNS Infections

There is mounting evidence that the brain injury during CNS infections is partially due to the resultant immune response, in addition to the destructive effects of the pathogen itself (146–149). CSF inflammatory cytokine levels are generally much higher in patients with CNS infections compared to those with neuroinflammatory disorders or brain tumors (150), and higher systemic and/or CSF levels of cytokines such as IL-1β, IL-6, and TNF have been variably associated with worse outcomes (151, 152). Acute cerebral edema is an infrequent but devastating complication of acute meningitis and encephalitis. In a study of 1038 children with acute encephalitis, 25 developed fulminant cerebral edema and 16 died (153). Similarly to CAR T cell patients, no consistent risk factors for cerebral edema during CNS infection have been identified to date. Although the pathophysiology is incompletely understood, it likely involves a vicious cycle of inflammatory host reaction with alteration of BBB integrity, additional local release of pro-inflammatory mediators, and dysregulation of cerebral blood flow (148).

### Hepatic Encephalopathy

Acute liver failure can cause rapidly progressive and fatal cerebral edema, which has been attributed to toxic effects of ammonia and other metabolites (154). While the underlying mechanisms of CAR T treatment related cerebral edema are different, and metabolic derangements such as hyperammonemia or hyponatremia are not typically observed in CAR T patients with cerebral edema, it may be instructive to understand whether there could be a final common pathway. Such overlaps in pathophysiology may involve dysfunction of glial water handling at the neurovascular unit, excitotoxicity, and energy failure (155). Inflammatory mediators may also modulate responses to metabolic challenges, and affect individual susceptibility to brain edema. For example, in mice, the induction of edema by ammonia injection requires expression of toll-like receptor 9 (TLR9), which is also required for the inflammatory response to bacterial meningitis (126). In rats, cerebral edema from hepatic encephalopathy induces microglial activation and upregulation of brain IL-1β, IL-6, and TNF, and treatment with minocycline decreases edema, microglial activation, and cytokine increases (156). Intracerebral injection of IL-1β and TNF in rats causes vasogenic edema (157), and anti-TNF antibodies reduce cerebral edema, possibly *via* reduction of matrix metalloproteinase production (158). At this point, it remains uncertain whether the cytokine increases observed in the brain during cerebral edema from liver failure play a causative role, or whether they simply represent a response to injury.

## Effects of the CAR T–Associated Inflammatory Secretome on Components of the CNS

**Figure 3:**
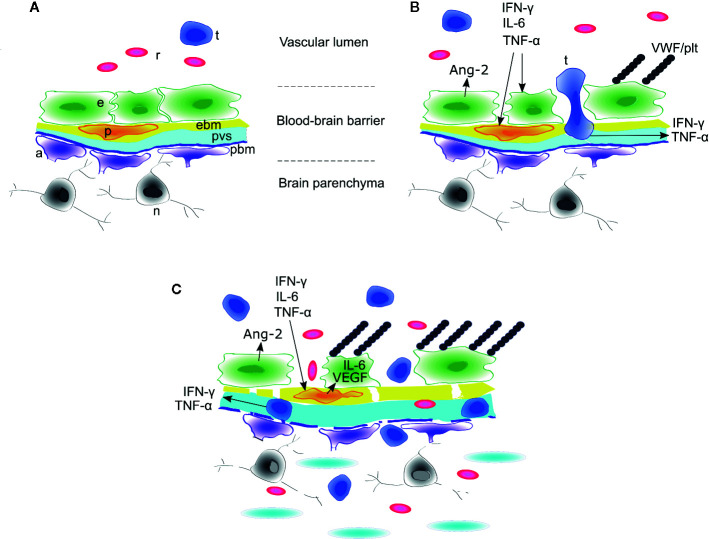
Model of blood-brain barrier disruption during ICANS. **(A)** Around cerebral microvessels, the normal blood-brain barrier (BBB) protects the brain parenchyma and neurons by regulating transit of leukocytes and cytokines into the perivascular space. **(B)** High concentrations of systemic cytokines such as IFNγ, IL-6, and TNFγinduce endothelial cell activation, resulting in release of angiopoietin-2 (Ang-2) and von Willebrand Factor (VWF) from endothelial Weibel-Palade bodies. VWF binds activated endothelium and sequesters platelets. Activated CAR T cells cross the endothelial barrier into the perivascular space and cerebrospinal fluid (CSF). **(C)** The concentrations of cytokines such as IFNγ and TNFγincrease in CSF due to transit from plasma across the disrupted BBB and/or secretion from CAR T cells or other immune cells undergoing diapedesis. High concentrations of IFNγ and TNF induce secretion of IL-6 and VEGF from brain vascular pericytes and induce pericyte stress, amplifying endothelial activation and BBB disruption. In the most severe cases, this may lead to breakdown of the parenchymal basement membrane and vascular disruption, with thrombotic microangiopathy, acute edema, red blood cell extravasation, microhemorrhage, and neuronal death. a, astrocyte end foot; e, endothelial cell; ebm, endothelial basement membrane; n, neuron; p, pericyte; pbm, parenchymal basement membrane; plt, platelets; pvs,perivascular space and cerebrospinal fluid; r, red blood cell; t, CAR T cell. Figure adapted with permission from (7).

Access of systemic molecules and cellular traffic to the CNS is tightly regulated by the blood-brain-barrier (63). In this section, we will examine two related but separate questions: 1) which components of the CNS mediate the physiologic response to systemic inflammatory signaling? and 2) how do systemic inflammatory mediators affect the function of specific components of the CNS? We will focus specifically on the neurovascular unit as the hypothesized primary locus of injury during ICANS (7). From the vascular lumen to the brain parenchyma, the neurovascular unit is comprised of endothelial cells connected by tight junctions, covering pericytes, endothelial basement membrane, and glia limitans with adjacent tightly tiled astrocyte endfeet (159) (**Figure 3**). Along the penetrating vessels, the endothelial side is separated from the glia limitans by a perivascular space that is contiguous with the CSF (160). When interpreting clinical data, it is important to recognize that multiple different barriers are in play when considering transport of signaling molecules from the blood to the CNS. Cells or molecules may traverse the choroid plexus barrier or meningeal vessels to enter the CSF, while still being unable to gain access *via* the microvascular BBB to enter the brain or spinal cord parenchyma (161). In ICANS, there is evidence of increased leakiness of blood-CSF barriers with transit of cells and inflammatory mediators into the CSF (see *CSF analysis* above). The presence of vasogenic or interstitial edema on brain imaging and histopathologic examination in patients with CAR T–associated cerebral edema suggests leakage of plasma around the vessels and, potentially, into the brain parenchyma (see *Imaging* above). We have less evidence of disruption of the various blood-CNS barriers in milder cases of reversible ICANS that are characterized primarily by acute language and/or cognitive dysfunction, where imaging is frequently normal. Thus, in addition to the BBB, we will also examine possible direct effects of cytokines on neuronal function, behavior and cognition.

### Brain Microvascular Endothelial Cells

Brain microvascular endothelial cells are a key regulator of access to the CNS by systemic inflammatory signals, as they mediate the central febrile response, synthesize additional inflammatory cytokines, and regulate adhesion and transit of peripheral immune cells. Brain endothelial cells express receptors for IL-1β, IL-6, and TNF (63). When challenged with inflammatory stimuli such as lipopolysaccharide or IL-1β, they synthesize prostaglandins (162), which then induce a febrile response by binding to thermoregulatory neuronal circuits in the hypothalamus (163). The brain endothelium also locally produces cytokines including IL-6, IL-8, GM-CSF, and TNF in response to pathogenic stimuli, where they can act in an autocrine fashion to accentuate the pro-inflammatory response and alter endothelial transporter function (164–167). Cytokines acting on brain endothelial cells regulate trafficking of immune cells by changes in expression of leukocyte adhesion molecules, which is additionally enhanced by the presence of fever (168). IL-β and TNF induce different patterns of adhesion molecule expression (169). Endothelial cell injury and systemic toxins or inflammation can cause endothelial activation, adhesion of leukocytes, platelet thrombus formation, and concomitant microvascular dysfunction (discussed in detail above under *Thrombotic Microangiopathy*).

Single-cell RNA sequencing data is now opening new perspectives on the diversity of brain endothelial cells, but data on changes in the endothelial transcriptome in inflammatory disease are just emerging (170–172). For example, a novel group of interferon-responsive brain endothelial cells was discovered by scRNAseq, whose functional role has yet to be studied (173). In summary, the interaction of systemic secreted mediators with the brain endothelium is an area that deserves intense further scrutiny in attempts to understand the pathophysiology of ICANS.

### Pericytes

The role of pericytes in neuroinflammation is an active field of investigation (174, 175). Pericytes cover the parenchymal side of the brain endothelial cells, where they play an important role in maintaining the integrity of the neurovascular unit (176). Pericytes secrete angiopoietin-1, which maintains endothelial quiescence. When exposed to angiopoietin-2, pericytes detach from vessels during angiogenesis (177). It is becoming increasingly clear that pericyte adhesion to the endothelium is required for regulating leukocyte diapedesis during systemic inflammation (178). For example, pericyte deficient mice have increased expression of the leukocyte adhesion molecules VCAM-1 and ICAM-1 on cerebral endothelial cells, leading to increased perivascular immune cell infiltration (179). After systemic LPS injection, pericytes detach from the basal lamina, thereby increasing BBB permeability to infiltrating cells (166). TNF and IFNγ induce secretion of IL-6 and VEGF from pericytes *in vitro*, which in turn may lead to endothelial activation (7). Although pericytes have not yet been directly implicated in the pathophysiology of CAR T neurotoxicity, their involvement deserves further study given the extensive crosstalk between pericytes, brain endothelial cells, and astrocytes in regulating the function of the neurovascular unit (80).

### Microglia

In their role as CNS resident myeloid cells, microglia play an important role in modulating CNS inflammation (180). Microglia are consistently activated during systemic inflammatory challenges such as sepsis. Microglial activation was noted to be widespread on histopathology of a patient with CAR T–associated cerebral edema (54), whereas it was limited to a perivascular distribution in another case (7). Microglia are activated *via* microbial or cellular injury signals binding toll-like receptors, which trigger cytokine release, microglial proliferation, and migration (181). *In vitro* studies show that TNF, IL-1β, and IFNγ can activate microglia as well, although this response may be weaker (182, 183). The study of the microglial response to systemic inflammation is complicated by rapid perivascular infiltration of monocytes/macrophages, which are difficult to distinguish from microglia by surface markers, and secrete additional proinflammatory cytokines that may cause further microglial activation (184). The role of activated microglia has been extensively studied in chronic neurodegenerative disorders such as Parkinson’s and Alzheimer’s diseases (185). Here, there is evidence a protective role in a brain that has been injured by primary degenerative processes, but also the possibility of additional injury *via* an accelerated inflammatory response (186). Single cell and single nucleus RNA sequencing are now rapidly advancing our understanding of microglial responses in CNS inflammation (187). A proinflammatory signature has been shown in a subset of human microglia (188, 189). It is not yet known whether this subclass has a distinctive response to systemic pro-inflammatory stimuli, but distinctive responses appear to occur during localized CNS inflammation. In mice, single cell RNA-seq shows upregulation of a interferon-response gene signature in a subpopulation of microglia after demyelinating brain injury (190, 191). In patients with multiple sclerosis, single nucleus RNAseq reveals marked expansion of microglia, with transcriptional changes toward a phagocytosing phenotype (192). Importantly, microglia retain memory of peripheral inflammatory stimuli by long-lasting epigenetically mediated changes in gene expression (193), which could contribute mechanistically to long-term neurologic sequelae in ICANS (8).

### Astrocytes

Astrocytes are likely key to understanding the CNS response to systemic cytokine elevation. In children with acute ICANS, CSF levels of glial fibrillary acidic protein (GFAP) increased significantly during acute toxicity when compared to pretreatment levels, indicating acute astroglial injury (8). In addition, astrocyte activation and gliosis are consistently seen on histopathology of patients who died from cerebral edema, or with chronic sequelae after ICANS (7, 8, 54). The astrocyte endfeet are a critical component of the neurovascular unit, and disturbances of solute handling by the astrocyte cause derangements in the exquisitely controlled interstitial electrolyte concentrations and subsequent neuronal excitotoxicity (194). Astrocyte swelling occurs during cerebral edema secondary to stroke, ischemic brain injury, acute liver failure, or blood hypoosmolarity, indicating a final common pathway. Astrocyte swelling is associated with energy failure and extracellular K+ and glutamate accumulation, and mediated by water uptake *via* aquaporin-4 channels (194, 195). *In vitro*, astrocyte swelling can be induced by IL-6, IL1β, TNF, or IFNγγ (196). The astrocyte endfeet also pose a key barrier to trafficking of immune effector cells into the brain parenchyma from the perivascular space. Systemic cytokines affect the astrocyte barrier; for example, IL-1β induces release of VEGF from astrocytes, which then disrupts tight junctions in the endothelial barrier and increases leukocyte extravasation (197). In addition, astrocytes produce pro- and anti-inflammatory cytokines in a complex response to various mechanisms of inflammatory brain injury (198, 199).

### Neurons

Although the brain parenchyma is well shielded from serum inflammatory mediators, neurons do develop dysfunction in response to systemic inflammatory stimuli. There are putative direct pathways that affect behavior in response to infection or inflammation *via* vagal afferents and/or the circumventricular organs (162, 200), although these are unlikely to result in the severe neurocognitive dysfunction that is seen in ICANS. In contrast, dysfunction of the neurovascular unit and alteration of glial solute handling likely play a very important role in the disturbance of neuronal function and possible excitotoxicity during systemic inflammation. Serum levels of IFNγ, IL-6, IL-8, IL-10, and IL-1RA were higher in children who developed febrile seizures, compared to levels in children with fever alone (201). When patients with ICANS develop seizures, these are often multifocal and without clear imaging correlates, indicating the presence of diffuse disturbance of neuronal function (49). In addition, CSF levels of glutamate and the excitotoxin quinolinic acid were increased in patients with ICANS compared to pre- and post-treatment levels, indicating the presence of excitotoxicity (12). There is compelling *in vitro* evidence that IL-1β and TNF directly alter neuronal excitability, but much less work has been done on other pro-inflammatory mediators implicated in ICANS (202). Systemic IL-1β or TNF release causes sickness behavior in mice, with decreased activity and food intake (203). In a study of patients hospitalized for any cause, those with higher serum levels of IL-6 or IL-8 were significantly more likely to develop delirium, supporting the concept that transient neurologic dysfunction during illness is cytokine related (204).

## Approaches to Modulating ICANS

Current research focuses on cytokine-based mechanistic interventions for ICANS. However, there is still no convincing evidence that blockade of a single specific inflammatory mediator can prevent or improve ICANS. Thus, corticosteroids remain the most commonly used intervention, as they reduce CNS edema and have broad immunosuppressive effects that reduce both cell-based inflammatory responses and secreted pro-inflammatory mediators. The possible benefit of treating ICANS must be balanced by the risks, chief of which is suppression of the CAR T cell response and impaired persistence, which could put the patient at increased risk of insufficient response or relapse. Because many cases of ICANS appear to be self-limited, and it is still not well known whether apparently reversible ICANS has any long-term sequelae, some investigators continue to treat mild ICANS with supportive care only (9). However, in light of the success of early and aggressive CRS treatment, others advocate early treatment of ICANS with immunomodulators to decrease the risk of rare but life-threatening cerebral edema (48, 205).

### Corticosteroids

Corticosteroids (glucocorticoids) have broad activity against production of many pro-inflammatory effectors, including IL-1β, IL-6, and TNF, by T cells (206), monocytes/macrophages (207), and vascular pericytes (208). They reduce expression of endothelial adhesion molecules, which limits tissue infiltration by circulating leukocytes (209). Additionally, they decrease production of VEGF, thereby reducing vascular permeability (210). Consequently, corticosteroids are a mainstay of ICANS treatment (14, 211), although there is unfortunately no high quality evidence that they are effective for this indication. Dexamethasone is most frequently used for ICANS, since it has excellent CNS penetration and is the standard treatment for vasogenic brain edema related to trauma or tumors (119, 212). While steroid use could theoretically impair CAR T cell proliferation *in vivo*, so far no adverse effect on anti-tumor efficacy has been definitively shown. Response rates were not affected by dexamethasone in a pediatric study of CD19-CAR T cell treatment for ALL (96). In adults with non-Hodgkin lymphoma, increased use of steroids and the IL-6 receptor blocker tocilizumab was associated with lower rates of CRS and neurotoxicity, without changes in tumor response or CAR T cell expansion (213).

Oral prednisone and methylprednisolone have well established efficacy and safety for neuroinflammatory disorders such as multiple sclerosis and acute demyelinating encephalomyelitis (214, 215). High-dose methylprednisolone is typically reserved for ICANS that is refractory to dexamethasone (7), since high-dose steroids may lead to more profound impairment of T cell proliferation and/or persistence (216). Given the relative rarity of their use, the effect of high dose steroids on CAR T cell proliferation or persistence has not been established. No clinical trials are currently registered to further investigate the role of steroids in the treatment of ICANS.

### IL-6 Blockade

Given the key role of IL-6 in the pathogenesis of CRS, IL-6 blockade with tocilizumab quickly emerged as standard of care for the treatment of CRS (217–219). Although high-quality evidence is not yet available, FDA approval for this indication was granted in 2017 after retrospective analyses showed sufficient likelihood of efficacy and safety (95, 96). There has been longstanding concern in the field that tocilizumab may worsen ICANS. The reason for this possible paradoxical effect is that the large size of the tocilizumab molecule likely prevents it from crossing the BBB. Since tocilizumab blocks the IL-6 receptor, and production of IL-6 may increase after receptor blockade, the CNS could theoretically be exposed to higher level of free IL-6 that can act unopposed (220, 221). A nonhuman primate study indeed showed that tocilizumab has poor penetration into the CSF when administered systemically, while direct administration into the intrathecal space was well tolerated and achieved levels similar to therapeutic serum levels (222). The published clinical evidence, however, is conflicting, and high quality randomized controlled trials are needed to resolve the question. In a study of 43 children and young adults treated with CD19-directed CAR T cells, the incidence of ICANS was similar between an early cohort that received tocilizumab only for dose limiting toxicities that did not resolve with standard medical intervention, and a follow-up cohort that received tocilizumab for persistent mild CRS (96). In addition, the timing of tocilizumab administration relative to the time of CRS onset did not change the severity of ICANS relative to CRS grade (8). In an open-label trial of prophylactic tocilizumab in adults with NHL, administration of tocilizumab to all patients on day 2 after CAR T cell infusion was associated with a decrease of grade ≥ 3 CRS, but increased incidence of grade ≥ 3 ICANS compared to an earlier cohort where tocilizumab was given only after toxicity developed (10, 53). However, a safety expansion cohort of the same FDA-approved CAR T product (axicabtagene ciloleucel) showed lower rates of grade ≥ 3 ICANS compared to earlier cohorts which used tocilizumab less frequently (213). Currently, the use of tocilizumab remains institution-dependent, with most using tocilizumab only for CRS but not as a first line therapy for ICANS (223). However, in clinical practice, CRS and ICANS are often present concurrently, leading to administration of tocilizumab while ICANS signs and symptoms are present. It will be crucial to examine outcomes rigorously when trying to balance the risk of CRS with the risk of ICANS.

To avoid the possibility of a paradoxical IL-6 increase in the CNS after IL-6R blockade, siltuximab has been proposed as an alternative IL-6 antagonist. Siltuximab binds the IL-6 molecule directly and blocks it from binding both soluble and membrane-bound IL-6R (220, 224). However, the evidence for its use in ICANS remains anecdotal, and it is typically reserved for tocilizumab-refractory CRS since it is not FDA approved for the indication. Siltuximab has been used in the setting of CRS and/or ICANS after CD19-CAR T therapy (225), and for neurologic adverse events in a patient who received EGFRvIII-targeted CAR T cells for glioblastoma (41). No clinical trials of siltuximab for CAR T cell patients are registered at this time.

### IL-1 Blockade

The role of IL-1 signaling in CRS and ICANS has been established in several preclinical animal models (90, 226), prompting consideration of IL-1 blockade for treatment of these toxicities. Anakinra is a recombinant IL-1 receptor antagonist (IL-1RA), which is FDA approved for the treatment of multiple rheumatologic disorders (227), but safety and efficacy in CAR T cell patients have not been established. It is theoretically attractive for treatment of ICANS because it crosses into the CSF and is effective against CNS inflammation (89). Several open label clinical trials are in planning or underway to evaluate the efficacy of anakinra for prevention of ICANS and/or CRS after CD19-CAR T cell treatment in adult patients. No blinded, randomized, or prospective pediatric studies are currently active. Registered trials include:

- NCT04150913: subcutaneous anakinra to be administered to patients with non-Hodgkin lymphoma on days 0–6 after CAR T cell infusion. Primary outcome measure is incidence of grade 2+ neurotoxicity;
- NCT04359784: subcutaneous anakinra to be given on days 0-13 after CAR T cell infusion. The primary outcome measure is absence of any grade CRS; ICANS grade is a secondary outcome measure;
- NCT04148430: cohort 1 will receive anakinra on days 2–10 or while fever is present, whichever is longer. If cohort 1 shows adequate disease response and neurotoxicity rates, the study will proceed to cohort 2, where anakinra is given from days 0–6, with dose escalation if fever or ICANS develop. The primary outcome measure is the rate of grade ≥ 3 neurotoxicity or any seizure; and
- NCT04205838: anakinra to be administered to all patients who develop ICANS of any grade, or grade ≥ 3 CRS. Primary outcome measures are feasibility and rate of grade ≥ 3 ICANS.

### GM-CSF Blockade

Based on animal studies indicating efficacy of GM-CSF blockade against CRS and neurotoxicity (94), the anti-GM-CSF antibody lenzilumab has been proposed as a rational therapy for ICANS (93). An open-label trial of lenzilumab for prevention of ICANS in adults receiving CD19-targeted CAR T cells is in planning, with a primary outcome measure of grade ≥ 2 neurologic events (NCT04314843). There is no published data yet in humans yet describing the effects of GM-CSF blockade for the treatment or prevention of ICANS.

### Other Therapeutic Approaches Targeting Secreted Mediators

Removal of pro-inflammatory mediators from the blood has been proposed for the management of refractory CAR T toxicities, similar to the use of plasma exchange in septic shock (228). Case reports describe resolution of CD19-CAR associated refractory CRS with or without ICANS after treatment with cytokine adsorption plus standard hemodialysis (229), plasma exchange (230), and hemofiltration not otherwise specified (231). An extracorporeal cytokine adsorption technology (CytoSorb) is currently being evaluated in a randomized controlled pilot study to reduce IL-6 levels in patients with CRS and/or ICANS (NCT04048434).

There are multiple other clinical trials underway that target cytokines in CAR T cell patients. The majority of these trials focus on CRS but not ICANS as a primary outcome measure, although we expect that results will be reported on ICANS as well. For example, an open-label study at Children’s Hospital of Philadelphia is underway to determine whether early administration of tocilizumab in patients with high tumor burden can reduce grade ≥ 4 CRS, while ICANS is not included as a predefined outcome measure of the study (NCT02906371) (232).

Another agent under investigation is defibrotide, an oligonucleotide derived from porcine DNA, whose mechanism of action is incompletely understood but likely involves modulation of endothelial cell-leukocyte interaction (233). Given the putative role of endothelial activation in the pathogenesis of ICANS (7), a clinical trial to evaluate its activity in preventing ICANS is currently enrolling (NCT03954106).

Dasatinib is a tyrosine kinase inhibitor that can reversibly shut off CAR T cell activity and reverse CRS in a mouse model (234). This occurs *via* halting of T cell proliferation and cytokine production (235), but it is not known whether this intervention can also decrease cytokine production by the myeloid compartment.

Other mechanistically directed therapeutic approaches may include angiopoietin-1 augmentation or platelet hypertransfusion to counteract endothelial activation and coagulopathy (7, 100). There is no published clinical experience yet with these approaches in CAR T cell patients. Prospective studies are also needed to clarify the role of supportive care and neuroprotective measures, the clinical utility of ancillary studies such as EEG and imaging, and safety and efficacy of interventions such as intrathecal chemotherapy and therapies to lower intracranial pressure (14).

## Discussion

CAR T cell related neurotoxicity is a novel syndrome that presents with a clinical spectrum ranging from reversible neurocognitive dysfunction, to severe neurologic disturbances such as seizures and coma, to rare but extremely serious cerebral edema. It is clearly associated with increased systemic cytokine levels and CRS, but there appears to be a missing pathophysiologic link. Not all patients with severe CRS develop ICANS, and not all patients with ICANS have CRS. It is possible that the common manifestations of ICANS, especially language disturbance, have a different mechanism than the more dangerous manifestations, particularly cerebral edema, but no consistent distinguishing parameters have yet been uncovered to explain why ICANS manifests differently in different patients. As we gain experience with CARs other than CD19-directed therapies, we will achieve increasing clarity whether different targets are associated with different toxicity profiles, or whether ICANS is truly a single syndrome that represents a final common pathway of neuroinflammation related to immune-effector cell engaging cancer therapies.

The current state of the field supports a strong association of IFNγ, GM-CSF, IL-6, IL-10, IL-15, and possibly other inflammatory signaling molecules with ICANS. Based on our rich and complex knowledge of neuroinflammation, one could make a plausible case for any of these as directly causative, or at least modulating the development of neurocognitive dysfunction. We have limited data from patients to elucidate immune cell behavior during CRS/ICANS, and consequently there is uncertainty on which immune cell subsets to target with therapeutic interventions. Single cell expression analysis of patient-derived immune effector cells during toxicity will provide further insights.

Despite the mounting support for a key role of pro-inflammatory cytokines in the pathogenesis of ICANS, there is no high-quality evidence in humans to help us understand whether cytokine blockade can prevent or alleviate neurologic symptoms. To solve this problem, a multi-pronged approach is needed. First, careful clinical phenotyping and comprehensive collection of outcome data will be required to define research questions. Next, dedicated cytokine analysis will need to be conducted to include putative neuro-active inflammatory mediators that have been incompletely characterized in ICANS, such as IL-1β signaling. Future studies need to be designed to increase the number of time points and compartments (serum and CSF) to better understand the effect of CRS on cytokine profiles. Based on the hypotheses generated from clinical data, relevant preclinical animal models can then be used for mechanistic studies and testing of novel therapeutic approaches to design future prospective clinical trials.

As CAR T therapy approaches the mainstream and the field’s focus is first and foremost on anti-cancer efficacy, it will be important to continue to build in investigations to closely monitor, understand and treat toxicities.

## Author Contributions

All authors conceptualized and wrote the manuscript. JG additionally performed literature and data analysis. All authors contributed to the article and approved the submitted version.

## Funding

JG received funding through the Child Neurology Career Development Program K-12 award (1K12NS098482-02).

## Conflict of Interest

JG is a consultant for Johnson & Johnson. RP is employed by the company Shape Therapeutics but declares no overlap of commercial interests with this work. WL and CT are listed as inventors on the patent, “Biomarkers and uses thereof for selecting immunotherapy intervention”. CT receives research funding from Juno Therapeutics, Nektar Therapeutics, AstraZeneca, and TCR2 Therapeutics; serves on the Scientific Advisory Boards for Precision Biosciences, Eureka Therapeutics, Caribou Biosciences, T-CURX, Myeloid Therapeutics, ArsenalBio, and Century Therapeutics; *ad hoc* advisory boards (last 12 months): Nektar Therapeutics, Allogene, PACT Pharma, Astra Zeneca, Amgen; holds stock/options in Precision Biosciences, Eureka Therapeutics, Caribou Biosciences, Myeloid Therapeutics, and ArsenalBio, and holds a patent licensed to Juno Therapeutics.

The remaining author declares that the research was conducted in the absence of any commercial or financial relationships that could be construed as a potential conflict of interest.

## Abbreviations

ALL, acute lymphoblastic leukemia; BBB, blood-brain-barrier; BCMA, B cell maturation antigen; CLL, chronic lymphocytic leukemia; CAR, chimeric antigen receptor; CRS, cytokine release syndrome; CSF, cerebrospinal fluid; CNS, central nervous system; CXCL10, C-X-C motif chemokine ligand 10, also known as interferon-γ-inducible protein 10 (IP-10), which is the name used by most studies cited here; EGFRvIII, epidermal growth factor receptor variant III; GM-CSF, granulocyte-macrophage colony-stimulating factor; IFNγ, interferon gamma; ICAM-1, intercellular adhesion molecule 1; ICANS, immune effector cell–associated neurotoxicity syndrome; IL, interleukin; IL-1RA, interleukin-1 receptor antagonist protein; LPS, lipopolysaccharide; MRI, magnetic resonance imaging; NHL, non-Hodgkin lymphoma; TNF, tumor necrosis factor, referred to in most studies cited here as TNFα; TNFR, tumor necrosis factor receptor; TIE2, Angiopoietin-1 receptor; VCAM-1, vascular cell adhesion protein 1.
